# Une cause rare de compression médullaire: l’angiolipome épidural rachidien

**DOI:** 10.11604/pamj.2017.26.109.8702

**Published:** 2017-02-28

**Authors:** Abderrazzak El Saqui, Mohamed Aggouri

**Affiliations:** 1 Service de Neurochirurgie, CHU Hassan II, Fès, Maroc

**Keywords:** Angiolipome, épidural, laminectomie, imagerie par résonance magnétique, rachis, Angiolipoma, epidural, laminectomy, Magnetic Resonance Imaging, spine

## Image en médecine

Une étudiante de 19 ans, hospitalisée au service de Neurochirurgie pour une lourdeur des deux membres inférieurs évoluant depuis deux mois chez qui l'examen clinique trouve une paraparésie permettant la marche avec aide (Grade D de FRANKEL), un syndrome pyramidal aux deux membres inférieurs avec une douleur provoquée à la palpation des épineuses de la partie moyenne du rachis dorsal. Les explorations paracliniques ont consisté à la réalisation d'une IRM médullaire en première intention (A,B) qui a mis en évidence un processus lésionnel intracanalaire, épidural postérieur, qui s'étend de la première à la huitième vertèbre thoracique et comprime fortement le cordon médullaire. La patiente a été opérée en urgence par voie postérieure avec résection totale d'un processus facilement clivable de la dure-mère et saignant au contact. Les suites opératoires immédiates étaient simples et l'étude anatomopathologique (C) a mis en évidence une prolifération composée par la juxtaposition de lobules d'adipocytes matures et de cavités vasculaires, concluant à un angiolipome de nature strictement bénigne. La patiente a complètement récupéré son autonomie trois mois en postopératoire. Les angiolipomes épiduraux rachidiens sont des tumeurs bénignes rares qui se voient surtout chez l'adulte jeune, qui se manifestent par un tableau de compression médullaire lente. L'imagerie par résonance magnétique constitue actuellement l'examen de choix dans l'exploration de cette tumeur. Cependant, le diagnostic de certitude reste histologique.Le traitement est toujours chirurgical, la voie d'abord est habituellement postérieure et l'exérèse est souvent complète.

**Figure 1:**
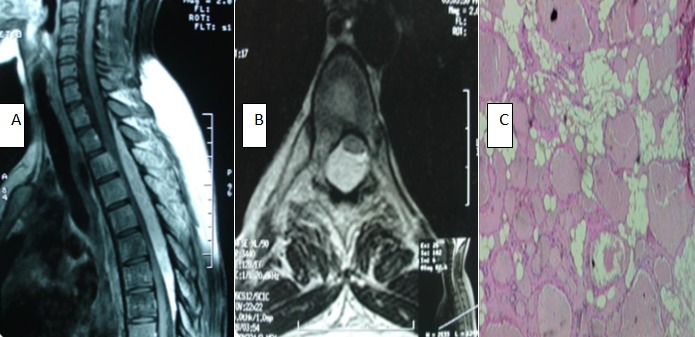
A) IRM rachidienne cervico-thoracique en séquence pondérée T1 montrant un processus épidural postérieur hétérogène, prenant le produit de contraste, étendu de D1 à D8, comprimant fortement la moelle. B) IRM rachidienne en coupe axiale T1 avec injection de Gadolinium montrant un processus épidural postérieur, refoulant la moelle qui est plaquée contre la face postéro-latérale gauche du corps vertébral. C) HES X 4, Angiolipome épidural; prolifération à double composante vasculaire et adipeuse

